# Are the users of social networking sites homogeneous? A cross-cultural study

**DOI:** 10.3389/fpsyg.2015.01127

**Published:** 2015-08-14

**Authors:** María-del-Carmen Alarcón-del-Amo, Miguel-Ángel Gómez-Borja, Carlota Lorenzo-Romero

**Affiliations:** ^1^Business Department, Universitat Autònoma de BarcelonaBarcelona, Spain; ^2^Business Department, University of Castilla-La ManchaAlbacete, Spain

**Keywords:** cross-cultural influence, social networking sites, latent international segmentation, user profiles, comparative study

## Abstract

The growing use of Social Networking Sites (SNS) around the world has made it necessary to understand individuals' behaviors within these sites according to different cultures. Based on a comparative study between two different European countries (The Netherlands versus Spain), a comparison of typologies of networked Internet users has been obtained through a latent segmentation approach. These typologies are based on the frequency with which users perform different activities, their socio-demographic variables, and experience in social networking and interaction patterns. The findings show new insights regarding international segmentation in order to analyse SNS user behaviors in both countries. These results are relevant for marketing strategists eager to use the communication potential of networked individuals and for marketers willing to explore the potential of online networking as a low cost and a highly efficient alternative to traditional networking approaches. For most businesses, expert users could be valuable opinion leaders and potential brand influencers.

## Introduction

A high degree of prosocial tendencies in human interactions has been revealed by behavioral economics research, suggesting a necessary shift from a model of selfish preferences toward social preferences—the assumption that people intrinsically are concerned about others' well-being (Caviola and Faulmüller, 2014).

From the marketing point of view, the importance of analysing the extraordinary evolution of new technologies is very useful to come to understand the evolution of consumer behavior in a cross-cultural context.

The spread of information and communication technologies has been growing so quickly that scholars have barely started to study its arrival, use, and effects on societies. Historians and other social scientists have the opportunity to examine the emergence of new and significant technologies and their effects while they are taking place—not decades or centuries after the event (Cortada, 2013). Information technologies and applications allow the definition of interactive spaces (i.e., the Internet) where all events and behaviors are registered and easily accessed. Moreover, it is possible to use new methodologies and tools within these environments to analyse these effects with a higher level of detail.

The new social medias' provide people with bodies that are combinations of embodied and technologically mediated actions. According to Jackson and Wang (2013), Social Networking Sites (SNS) “consists of a representation of each user (often called a profile), their social links, and a variety of additional series. Most SNS are web-based and provide a means for users to interact over the Internet, such as postings, e-mail and instant messaging. SNS may contain category places (such as school year), a means to connect with friends (usually with self-description pages) and a recommendation system linked to trust” (p. 911). SNS have supposed an essential cultural and technological revolution for individuals and businesses. In the case of business, these changes are obvious and are growing daily, at least for three fundamental reasons. First, individuals themselves can influence each other through their conversations on these networks (Kozinets, 2002). Second, SNS can be used to research attitudes, behaviors and profiling the members who interact in these networks (Kozinets, 2002; Ridings et al., 2002; Pitta and Fowler, 2005; Ackland, 2009). Finally, organizations could create social networking services, that can favor emotional connections and feelings of belonging for consumers, where individuals perceive the company as being concerned for their needs, and enabling a higher engagement and loyalty (Koh and Kim, 2004). For this reason, the analysis this type of networks and their applicability is particularly relevant.

The comparative approach allows the comprehension of the effects of factors associated with a specific trait or behavior across a range of social environments (Quinlan and Quinlan, 2007). Earlier studies have shown that cultural differences between countries have an impact on the effectiveness and efficiency of implementation and acceptance of information and communication technologies (ICT) in general and SNS in particular (Srite, 1999, 2006; Papacharissi, 2011).

National culture affects the technology acceptance (i.e., computers, the Internet, mobiles) through its impact on some key variables associated with the implementation process (Baron and Hard af Segerstad, 2010; Westlund, 2010; Jensen and Heles, 2011; Smith, 2011). Within this frame, De Brujin (2014) explores how connecting technologies have changed the social dynamics of African mobile communities and focuses on the changes in social hierarchies that are related to the access to mobility and connecting technologies. The culture of a country represents a set of shared values which influence attitudes, social perceptions, preferences and behavioral responses (Zhou et al., 2007; Baron and Hard af Segerstad, 2010; Westlund, 2010; Hemert et al., 2011; Smith, 2011). ICT researches have often referred to the cultural dimensions developed by Hofstede (1984), one the most influential scholar within the cross-cultural research arena (e.g., Steers et al., 2008; Dinev et al., 2009; Aldás et al., 2010; Lu, 2011).

According to different sources (e.g., ComScore Data Mine, 2013; GWI, 2014) SNS now reach more than 80%, representing more than 1.2 billion users around the world. SNS are ranked as the most popular content category in worldwide engagement, accounting for 19% of all time spent online. At this moment, people around 1,7 h a day on social networking sites.

Once the relevance of SNS have been confirmed, both for business and individuals, the general purpose of this study is to contribute to the existing knowledge body about the interaction behavior of SNS users, and to analyse the influence of culture on SNS acceptance and use. To that end, we will focus on two European countries with clearly different usage levels of SNS, and with distinct cultural dimensions according to Hofstede (1984). More specifically, this study focuses on analysing the differences in use, motivations, preferences, and attitudes regarding SNS between Dutch and Spanish users.

Spain and The Netherlands represent two of the largest European countries in SNS adoption. According to Eurostat data (2014), 92% of Dutch and 66% of Spanish citizens regularly (i.e., every day or almost every day) use the Internet (Eurostat, 2014). However, in terms of adopting SNS, there is not such a large difference between both countries. In December 2012, 85.1% of Dutch Internet users and 84.6% of Spanish Internet users were also SNS users. In addition, Spanish people spend much more time on SNS, regardless of age, than Dutch users (ComScore Data Mine, 2013). Thus, while both are European countries, their preferences and behavior, regarding SNS, are different, hence the importance of studying the different elements influencing the acceptance of SNS in both countries, and of exploring the characteristics and variables which both countries consider most important when using a social network. A comparison of Spain and The Netherlands will let SNS providers know the socio-demographic and cultural variables which add the most value for users in each country, and which should be adapted to consumers to increase their use.

The huge growth of SNS has strongly attracted the interest of business strategists and marketing practitioners; publicity, research papers, and journal special issues around the subject are also increasing. Despite this growing interest of researchers in SNS as part of the marketing strategy, little academic attention has been so far placed on the nature and behavior of the online SNS user. This study is a step in the direction of mapping the SNS users by identifying market segments and profiles on the basis of socio-demographics, motivations and behavioral responses. Therefore, the purpose is to build a taxonomy of users as the first step to come to understand the behavior of this category of consumers. Segmentation in general and SNS segmentation in particular lead to various benefits for business (Dibb, 2005). Segmentation provides the basis for a competitive advantage in the market and a greater customer satisfaction and higher level of customer loyalty, by dealing with diverse customer needs and by focusing resources on particular customer groups with relatively homogeneous requirements.

What is more, according to Sheth and Parvatiyar (2001) and Adams (2011), due to the increasing spread of technology and media and the increasing cultural permeability, homogeneous groups of consumer segments that transcend country boundaries are turning out to be relevant as target groups. Therefore, not only the different behavior patterns within each country will be analyzed (i.e., if there is heterogeneity or homogeneity among the users in each country), but also the potential similarities and differences between both countries. This research provides useful insights about the demographics, attitudinal and behavioral responses of the online networked consumer and contributes as a basis for future research directions. It also provides practitioners basic and essential information into the behavior of networked Internet users, as a starting point to engage SNS in their marketing strategy.

## Theoretical background

### Segmenting social networking sites markets: an international perspective

The diversity of members within social networks affects their behaviors within these informational environments (Wang and Fesenmaier, 2003). One of the characteristics that significantly affect their behaviors is personality and, in particular, these traits related with sociability, selflessness or extroversion. These people tend to be expressive, with common sense, high self-esteem, competent, with a low need for approval and high moral development (Straub, 1978; Rushton, 1981; Aronoff and Wilson, 1984). Also, they often need to feel useful, help others, have responsibilities, and earn a place where they belong, among other things (Wang and Fesenmaier, 2003). These people are very sociable, enjoy helping others, are fun and creative, and have high self-esteem (Wilson and Petruska, 1984). It is to be expected that members who are active and generous in their offline life will also be so in their online activities.

As well as personality, another characteristic affecting the contribution level is the general participation within SNS (Wang and Fesenmaier, 2003). Members can participate in different ways and at different levels. Some of them participate in a passive way (i.e., without contributing anything) while others take a more active part by interacting, communicating and sharing knowledge. Previous research has shown that the participation patters affect the users' motivations to attend to, understand and process information (Greenwald and Leavitt, 1984; Zaichkowsky, 1985). This is because participation has an important effect on peoples' interest (Richins and Bloch, 1986).

Age is a personal characteristic with high influence in social media contexts. Güroğlu et al. (2014) show that teenagers are getting increasingly better at incorporating social context into decision-making. Their findings further highlight the role of friendships as a significant social context for the development of prosocial behavior in early adolescence.

Individuals also differ in their attitudes to the SNS and in their behavior when they use them. Ofcom (2008) classified users of SNS into five distinct groups, based on their behaviors and attitudes: Alpha Socialisers (individuals that use SNS intensely, brief periods to flirt, meet new people or be entertained), Attention Seekers (users look for the attention and comments of others, uploading striking photos to their social network), Followers (users tend not to be early adopters of SNS, but join these sites to keep up with the activities of their real-life contacts), Faithfuls (people use social networks to recover past friendships, more than to make new contacts) and Functionals (occasional users, with short visits).

Campbell et al. (2014) analyse how consumers may be segmented with respect to their attitudes and reactions toward social network marketing, including psychological, economic, and socio-demographic covariates in a latent-class analysis. They identify five segments -Passive, Talkers, Hesitant, Active, and Averse—along with significant covariates, such as information search, convenience, entertainment, age and gender that predict membership. From supply perspective, Chung et al. (2015) obtain four distinct segments of consumers who support social ventures (social observers, active contributors, social connectors, and moderate contributors) based on three dimensions of social media site usage: creating content, connecting with others, and control over the user experience. Four segments of consumers show significant differences in supporting behaviors to create social ventures.

Despite the increasing importance of international market segmentation for marketing as a discipline (in particular, for international marketing), the relative importance as research topic in marketing literature remains low. Of course, a series or papers devoted to international market segmentation have appeared during the last three decades (e.g., Luqmani et al., 1994; Hofstede, 2001). Nevertheless, their number and scope are still astonishingly small in comparison to publications devoted to issues in domestic market segmentation (Bauer, 2000; Steenkamp and Hofstede, 2002; Larsson and Moe, 2012).

An international segmentation study carried out by Bastian et al. (2008) supports the opinion of Kale and Sudharshan (1987) and Bauer (2000) who pointed out some disadvantages of integral market segmentation, such as the impossibility of providing information on regional/national segments and of describing national peculiarities in the media usage and point of purchase choice behavior of consumers, as well as estimating national sizes of transnational segments in a biased way. The authors were convinced of that additive intranational market segmentation can overcome these issues. In general terms, it can be concluded that conducting additive intranational market segmentation should be preferred when identifying transnational segments. Hofstede et al. (2010) also carried out a cultural clustering of states that closely followed the administrative division of the Brazil country into five regions, obtaining remarkable differences between regions including other specific characteristics more meaningful to the analyzed region.

### Main differences in the adoption of new technologies and use of SNS. the case of Spain-the Netherlands

Hofstede (1980) is the starting reference point to evaluate the effects of differences in national cultures. His work provides an empirical base, and numerical evaluations of cultural dimensions for a large number of countries, namely power distance (the extent of inequality between people and the degree in what is considered normal by the population), individualism (the degree to which individuals prefer to act as individuals and not as members of groups), masculinity (the degree to which values considered as masculine such as performance, success and competence dominate among people), uncertainty avoidance (deals with a society's tolerance for uncertainty and ambiguity), and long-term orientation (how every society has to maintain some links with its own past while dealing with the challenges of the present and future). However, even though many researchers use Hofstede's cultural dimensions to explore the impact of cultural differences on the adoption and use of technologies, different results have been obtained for the dimensions of the culture being analyzed, depending on the technology studied.

**Figure 1:**
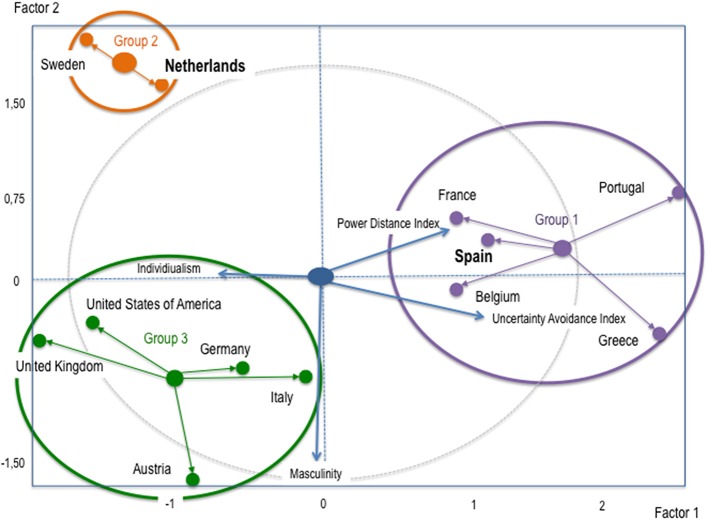
**Positioning countries by cultural dimensions**. Source: Grande (2004, p. 109).

On this subject, Grande (2004) carried out a cultural positioning of some countries based on the values they take in the cultural dimensions proposed by Hofstede (2001) of power distance, individualism, masculinity, and uncertainty avoidance. That is, for all the dimensions proposed by Hofstede (2001), except for long term orientation, which is covered for a few countries only, including The Netherlands but not Spain, so we will not analyse this dimension. Grande (2004, p. 109) identified three groups of countries in which Spain and The Netherlands are included (Figure 1):

Group 1: Latin European countries, concretely Belgium, France, Spain, Portugal, and Greece, characterized by high power distance and high uncertainty avoidance, are located in the right-hand part of the positioning map. It should be pointed out that the values in these two dimensions are higher when the country is further to the right on the map.
Group 2: Anglo-Saxon countries, concretely the USA, England, and Austria. They are more individualist, while also having lower power distance and low uncertainty avoidance, and are located to the left of the map. They are also very masculine cultures, being located in the lower part of the map (a more negative projection over the axis indicates more masculinity).
Group 3: Group consisting of The Netherlands and Sweden. They are characterized by individualism (at the same time, they have less power distance and low uncertainty avoidance) and low masculinity (high femininity).

In either case, there have been not many cross-cultural research addressing specifically the differences between Spain and The Netherlands. Grande (2004) observes clear differences between Spain and The Netherlands, mainly in the degree of individualism, power distance and uncertainty avoidance. Table 1 shows the numerical values of cultural dimensions in Spain and The Netherlands. In each of these dimensions, we have categorized Spain and The Netherlands as relatively high or low compared to the other. Thus, Spain can be classified as relatively high in the dimensions of power distance, masculinity and uncertainty avoidance, and relatively low in individualism, long term orientation and indulgence. In contrast, The Netherlands can be classified as relatively low in the dimensions of power distance, masculinity and uncertainty avoidance, and relatively high in individualism, long term orientation and indulgence. As well as the numerical values of each dimension in Spain and The Netherlands, Table 1 also shows in brackets the absolute position of each country in relation to the others for each dimension. As an additional value, in the last column we have reflected the difference in scores between Spain and The Netherlands for each dimension as well as differences in ranking positions.

**Table 1:** **Values of cultural dimensions proposed by Hofstede**.

**Dimension**	Spain Score^*^ (position)	**The Netherlands Score (position)**	**Difference Score (position)**
Power Distance Index (PDI)^**^	57 (41)	38 (55)	19 (14)
Individualism (IDV)^**^	51 (28)	80 (5)	−29 (23)
Masculinity (MAS)^**^	42 (48)	14 (67)	28 (19)
Uncertainty avoidance Index (UAI)^**^	86 (18)	53 (48)	33 (29)
Long term orientation (LTO)^***^	48 (44)	67 (22)	−19 (22)
Indulgence vs. restraint (IVS)^***^	44 (46)	68 (16)	−25 (30)

*Source: Based on http://www.geert-hofstede.com/ and http://www.geerthofstede.nl*.

* 1–100 scale;

** Based on 78 countries;

*** *Based on 96 countries*.

**Figure 2:**
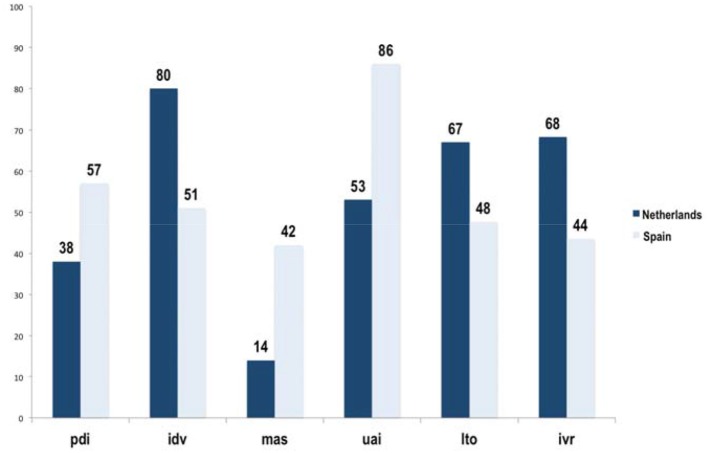
**Differences between Spain and The Netherlands**. Source: Hofstede (2015). http://www.geert-hofstede.com.

Therefore, power distance is greater in Spain than in The Netherlands, meaning that Spanish people more readily accepts an unequal distribution of power. Also, this is the dimension with the least difference between both countries together with long term orientation, which are located near the midpoint (Figure 2).

The Netherlands scores higher than Spain on individualism (a value of 80 compared to 51), taking fifth place. However, Spain is near the midpoint on the scale and is ranked 28th of all the countries in the study. The high level of individualism of The Netherlands is an indicator that this society has more individualist attitudes and fewer cohesive bonds with others. Dutch people has more self-confidence and is more independent. In the case of Spain, it may be said that relationships are closer, people have stronger bonds with other members of society, and there is greater group cohesiveness.

The score for masculinity is higher in Spain than in The Netherlands (42 compared to 14), although both countries are below the average score of 50. Therefore, we observe that Spain is already moving on from its formerly masculine character and is gradually acquiring feminine characteristics (Grande, 2004). Compared to the other countries, Spain ranks 48th, while Netherlands is 67th out of 69, so that we can affirm that Dutch culture is more feminine. Thus, we can state that people in Spain tend to be informal and goal oriented, while the Dutch value punctuality, voluntary associations, progress and innovation (Grande, 2004).

Regarding uncertainty avoidance, we observe that Spain scores close to the top of the uncertainty avoidance scale (a value of 86), ranking 19th, while The Netherlands scores close to the midpoint of the range (a value of 53) and ranks 48th. It should also be remarked that this is the dimension with the higher difference between both countries. Thus, we can state that the higher uncertainty avoidance showed by Spanish people means a higher willingness adopt regulations and laws which are intended to minimize uncertainty levels. They attempt to control everything in order to avoid the unexpected. As a result of high uncertainty avoidance, the society is more resistant to change (Hofstede, 2001). In fact, according to Hofstede (2015), “in the editions of Geert Hofstede's work since 2001, such as the most recent 3rd edition from 2010, scores are listed for 76 countries and regions, partly based on replications and extensions of the IBM study on different international populations. Since culture only changes very slowly, the scores can be considered up to date.” In this research, we are using the base culture data for 2010.

With reference to the long term orientation dimension, Spain scores lower than The Netherlands (48 vs. 67) meaning that Spanish culture tends to maintain traditions and norms and more reluctant to social changes. By contrary, Dutch culture has a more pragmatic orientation with easily adaptable traditions to changing conditions, strong propensity to save and invest and a high perseverance in achieving objectives.

Finally, the indulgence score for Spain is lower than for The Netherlands (44 vs. 68). These results mean that Spain can be defined as a restrained society with tendency to pessimism and where people think that their actions are restrained by social norms. The Netherlands is a clear example of indulgence, with a higher willingness to realize their needs and desires regarding enjoying life and having fun. They are more optimistic and place a higher importance on leisure time.

From another point of view, Fischer et al. (1999) and Rodríguez Mosquera et al. (2002), using the Schwartz's (1992) questionnaire of values, have also analyzed differences in cultural values between Spain and The Netherlands. Although with honor as a research topic, results from their research are consistent and can provide additional evidence about the cultural differences between both countries. Values such as ambition, capability, freedom, helpfulness, independence, moderation, responsibility and self-discipline are higher for the Dutch culture, while values such as family security, humility, honor, respect for parents and elderly, respect for tradition, social power and social recognition are revealed higher for Spanish people. These results are quite coherent with Hofstede's general results. Spain can be seen as more masculine, traditional, socially oriented and risk averse culture than the Dutch culture.

## Method

### Participants and procedure

This research was based on an online survey to SNS users in Spain and The Netherlands that was carried out in 2009–2010 to people with ages between 16 and 74. For both samples, participants who had account and used the SNS frequently were chosen to be analyzed in. The final sample size was of 799 SNS users (399 from Spain and 400 from The Netherlands), and was selected using a non-probabilistic quota sampling method.

This study was carried out in accordance with the current legal and ethical recommendations about privacy of personal data. During the whole process the ICC/ESOMAR International Code on Market and Social Research practices and norms were also taken into account. Specifically, questions regarding handling personal data and personal identifiers and other privacy policy issues recommendations included in the ICC/ESOMAR code.

In this method, a population is first segmented into mutually exclusive sub-groups; then, the number of sampled units in each category is specified, to assure that all the sub-groups are adequately represented in the sample (Lim and Ting, 2012). Our objective was to assure that the different population sub-groups were represented in the sample in the same way (i.e., similar percentage) in which they are present in the population regarding the sex, age, and residence.

### Data analysis: a latent segmentation approach

To develop the segmentation and profiling of the SNS users a latent segmentation methodology, using the Latent Gold 4.5 statistical software was used. This methodological approach allows the assignation of individuals to the segments based on their probability of belonging to the different clusters, breaking with the restrictions of deterministic assignment inherent to non-hierarchical cluster analysis (Dillon and Kumar, 1994). Latent segmentation allocates the subjects to different segments under the assumption that the data stems from a mixture of distribution probabilities or, in other words, from various groups or homogenous segments that are mixed in unknown proportions (McLachlan and Basford, 1988). Unlike cluster analysis methods, which are data-driven and model-free, latent class analysis is model-based, true to the measurement level of the data, and can yield sounder results for the explanation of consumer behavior (Wedel and Kamakura, 1999).

Traditional clustering methods, like K-means, have been reported to be useful in the social sciences. Nevertheless, it is often difficult for such methods to handle situations where clusters in the population overlap or are ambiguous (Bolin et al., 2014). The advantage of latent class models is that they allow the inclusion of variables with different measurement scales (metric or not). Also, the models can normally incorporate independent variables that may be used to describe (rather than to define or measure) the latent classes. These exogenous variables are known as covariates or grouping variables (McCutcheon, 1987; Hagenaars, 1993; Vermunt and Magidson, 2005).

### Measures

The variables that we used as indicators for the cluster analysis were based on the frequency with which users engage in different activities within the SNS on a four point scale (i.e., never, rarely, sometimes or frequently), to avoid the mid-point and force to form an opinion. On the other hand, different social-demographic characteristics (i.e., gender and age) were introduced as covariates to profile the resulting segments. Other covariates were experience with SNS, frequency of participation, time spent browsing in SNS, location, number and type of contacts, number of SNS used, and main motives for using these websites. Based on the answers of the different individuals, with regard to these questions, we obtained different grouping patterns that fulfill the principles of maximum internal coherence and maximum external differentiation (see Table 1).

## Results: a typology of Spanish and Dutch users of social networking sites

Once the latent segmentation approach was applied, the first step consisted of selecting the optimum number of segments. Latent Gold estimates models from one (i.e., no heterogeneity) up to eight (i.e., maximum heterogeneity). Tables 2, 3 show the estimation process summary and the fit indexes for each of the eight models in Spain and in The Netherlands, respectively.

**Table 2:** **Summary of models' results for Spain**.

**Number of conglomerates**	**LL**	**BIC(LL)**	**Npar**	**L^2^**	**Class.Err**.	**E_*s*_**	**R^2^**
1-Cluster	−9957.2188	20273.7752	60	19914.4376	0.0000	1	1
2-Cluster	−8691.7527	18024.3243	107	17383.5054	0.0132	0.95	0.96
3-Cluster	−8332.3777	17587.0555	154	16664.7554	0.0210	0.94	0.95
**4-Cluster**	−**8087.0424**	**17377.8660**	**201**	**16174.0847**	**0.0310**	**0.93**	**0.93**
5-Cluster	−7951.6918	17388.6460	248	15903.3836	0.0317	0.94	0.93
6-Cluster	−7828.8586	17424.4607	295	15657.7171	0.0421	0.93	0.92
7-Cluster	−7717.7561	17483.7370	342	15435.5122	0.0286	0.95	0.94
8-Cluster	−7663.3435	17656.3930	389	15326.6870	0.0256	0.96	0.95

*LL, log-likelihood; BIC, Bayesian information criterion; Npar, number of parameters; L^2^, likelihood-ratio statistic; Class.Err., classification error; Es, entropy R-squared; R^2^, Standard R-squared. Boldface indicates the selected model*.

**Table 3:** **Summary of models' results for The Netherlands**.

**Number of conglomerates**	**LL**	**BIC(LL)**	**Npar**	**L^2^**	**Class.Err**.	**E_*s*_**	**R^2^**
1-Cluster	−8388.3321	17136.1520	60	16776.6642	0.0000	1	1
2-Cluster	−7483.5478	15614.1738	108	14967.0956	0.0182	0.93	0.94
**3-Cluster**	−**7256.9279**	**15448.5242**	**156**	**14513.8558**	**0.0327**	**0.91**	**0.91**
4-Cluster	−7114.0080	15450.2748	204	14228.0160	0.0406	0.91	0.91
5-Cluster	−7022.5604	15554.9699	252	14045.1209	0.0489	0.91	0.90
6-Cluster	−6923.1160	15643.6714	300	13846.2321	0.0444	0.93	0.92
7-Cluster	−6845.0906	15775.2109	348	13690.1812	0.0261	0.96	0.95
8-Cluster	−6780.0176	15932.6552	396	13560.0352	0.0266	0.96	0.95

*LL, log-likelihood; BIC, Bayesian information criterion; Npar, number of parameters; L^2^, likelihood-ratio statistic; Class.Err., classification error; Es, entropy R-squared; R^2^, Standard R-squared. Boldface indicates the selected model*.

The fit of the model was evaluated with the Bayesian Information Criterion (BIC), which allows the identification of the model with the least number of classes that best fits to the data. BIC statistic has been used to select the optimum number of segments, since it is especially useful in comparing models (Magidson and Vermunt, 2001).

Based on the BIC equation

$$\begin{array}{l} \text{BIC}=-2\text{LL}+\text{ln}\left(\text{N}\right)\times \text{M} \end{array}$$

where LL is log-likelihood, N is sample size, M is number of parameters, and ln is natural logarithm, the computation for both countries is the following:

$$\begin{array}{l} \text{In the case of Spain }\left(\text{fourth cluster}\right):\\ 17,377.8660=-2\;\left(8087.0424\right)+\text{ln }\left(399\right)\times 201\\ \text{In the case of The Netherlands }\left(\text{third cluster}\right):\\ 15,448.5242=-2\;\left(7256.9279\right)+\text{ln }\left(400\right)\times 156 \end{array}$$

This statistic (information criteria) weight fit and parsimony by adjusting the LL to account for the number of parameters in the model. The lowest BIC value was considered as the best model indicator (Vermunt and Magidson, 2005).

In the case of Spain sample, the best alternative was reflected in a final solution of four different user groups. In the case of The Netherlands sample, the BIC is minimized in three groups.

The Model Fit likelihood ratio chi-squared statistic (L^2^) can be interpreted as “indicating the amount of the observed relationship between the variables that remains unexplained by a model; the larger the value, the poorer the model fits the data and the worse the observed relationships are described by the specified model” (Vermunt and Magidson, 2005, pp. 107–108). Moreover, the entropy statistic (Es) and R^2^ are near 1.

In addition to that set forth in Tables 2, 3, we have considered the Wald statistic, to evaluate the statistical significance within a group of estimated parameters (see Table 4). For all the indicators, in Spain and in The Netherlands, we obtained a significant *p*-value associated with the Wald statistic, which corroborate that each indicator significantly discriminates between clusters (Vermunt and Magidson, 2005).

**Table 4:** **Cluster profiles obtained (indicators)**.

	**Introvert**		**Novel**	**Versatile**		**Expert-communicator**		**Wald**		***p*****-value**		**R**^**2**^	
	**SP^†^**	**NL^‡^**	**SP**	**SP**	**NL**	**SP**	**NL**	**SP**	**NL**	**SP**	**NL**	**SP**	**NL**
Size	18.6%	41.3%	25.2%	36.2%	47.4%	19.9%	11.3%						
**Indicators:**													
**SHARE OR UPLOAD PHOTOS**													
Never	**0.443**	**0.352**	0.068	0.050	0.073	0.000	0.180	99.9	87.8	<0.001	<0.001	0.4	0.3
Rarely	0.315	**0.347**	0.191	0.165	0.227	0.005	0.252						
Sometimes	0.220	**0.282**	**0.530**	**0.535**	**0.578**	0.174	0.427						
Frequently	0.022	**0.019**	0.211	0.249	0.122	**0.821**	**0.140**						
**COMMENT ON FRIENDS' PHOTOS**													
Never	**0.468**	**0.661**	0.025	0.032	0.264	0.000	0.018	124.2	80.4	<0.001	<0.001	0.5	0.4
Rarely	0.317	0.265	0.129	0.147	**0.358**	0.001	0.117						
Sometimes	0.206	0.071	**0.640**	**0.640**	0.328	0.121	**0.507**						
Frequently	0.009	0.003	0.205	0.180	0.049	**0.878**	0.357						
**COMMENT ON WHAT THE PEOPLE THEY FOLLOW DO/SAY**													
Never	**0.571**	**0.773**	0.096	0.054	**0.240**	0.000	**0.017**	124.0	88.5	<0.001	<0.001	0.5	0.4
Rarely	0.266	**0.188**	0.208	0.160	**0.324**	0.000	**0.099**						
Sometimes	0.154	**0.038**	**0.558**	**0.589**	**0.364**	0.066	**0.480**						
Frequently	0.008	**0.001**	0.137	0.197	**0.071**	**0.933**	**0.404**						
**BROWSE ACROSS SNSs AND THEIR USERS' PROFILE**													
Never	**0.710**	**0.903**	0.281	0.200	**0.603**	0.039	0.232	74.5	59.4	<0.001	<0.001	0.3	0.3
Rarely	0.217	0.089	0.291	0.263	0.266	0.119	0.285						
Sometimes	0.065	0.008	**0.294**	**0.340**	0.113	0.358	**0.337**						
Frequently	0.009	0.000	0.134	0.197	0.018	**0.484**	0.146						
**UPDATE PROFILE**													
Never	0.430	0.284	0.069	0.027	0.042	0.002	0.002	97.9	80.1	<0.001	<0.001	0.4	0.3
Rarely	**0.452**	**0.544**	0.370	0.252	0.370	0.058	0.089						
Sometimes	0.113	0.167	**0.476**	**0.552**	**0.523**	0.452	**0.579**						
Frequently	0.004	0.004	0.086	0.169	0.065	**0.488**	0.330						
**SEND PRIVATE MESSAGES**													
Never	0.268	0.209	0.018	0.022	0.028	0.000	0.004	98.6	64.9	<0.001	<0.001	0.4	0.2
Rarely	0.337	0.317	0.113	0.127	0.135	0.003	0.043						
Sometimes	**0.364**	**0.413**	**0.611**	**0.616**	**0.569**	0.167	0.442						
Frequently	0.031	0.060	0.258	0.234	0.268	**0.830**	**0.512**						
**SEND PUBLIC MESSAGES**													
Never	**0.516**	**0.650**	0.098	0.067	0.284	0.000	0.126	121.1	64.3	<0.001	<0.001	0.5	0.2
Rarely	0.276	0.227	0.207	0.176	0.262	0.001	0.195						
Sometimes	0.199	0.105	**0.590**	**0.620**	**0.320**	0.127	**0.398**						
Frequently	0.009	0.017	0.104	0.136	0.135	**0.872**	0.281						
**LABEL FRIENDS IN PICTURES**													
Never	**0.790**	**0.947**	0.240	0.190	**0.663**	0.008	0.261	93.1	63.3	<0.001	<0.001	0.4	0.3
Rarely	0.178	0.051	0.322	0.302	0.266	0.059	**0.336**						
Sometimes	0.030	0.001	**0.326**	**0.361**	0.058	0.324	0.236						
Frequently	0.002	0.000	0.112	0.147	0.013	**0.609**	0.167						
**OBTAIN INFORMATION OF INTEREST**													
Never	**0.390**	**0.634**	0.264	0.062	0.236	0.006	0.076	81.2	71.0	<0.001	<0.001	0.3	0.3
Rarely	0.300	0.236	0.288	0.162	0.249	0.042	0.150						
Sometimes	0.261	0.108	**0.354**	**0.475**	**0.324**	0.343	0.368						
Frequently	0.049	0.022	0.094	0.301	0.190	**0.609**	**0.405**						
**DOWNLOAD APPLICATIONS**													
Never	**0.718**	**0.922**	**0.698**	0.179	**0.521**	0.169	0.219	83.1	63.1	<0.001	<0.001	0.3	0.3
Rarely	0.241	0.072	0.255	**0.361**	0.299	**0.355**	**0.293**						
Sometimes	0.038	0.005	0.044	0.344	0.154	0.352	0.352						
Frequently	0.002	0.000	0.003	0.116	0.026	0.124	0.136						
**DOWNLOAD GAMES**													
Never	**0.860**	**0.918**	**0.765**	0.298	**0.756**	0.364	**0.506**	66.7	32.6	<0.001	<0.001	0.2	0.1
Rarely	0.128	0.065	0.202	**0.371**	0.141	**0.372**	0.185						
Sometimes	0.011	0.015	0.032	0.276	0.083	0.227	0.215						
Frequently	0.001	0.001	0.001	0.055	0.019	0.037	0.094						
**LOOK FOR FRIENDS**													
Never	**0.645**	0.215	**0.334**	0.142	0.052	0.111	0.012	67.9	48.1	<0.001	<0.001	0.2	0.2
Rarely	0.242	0.301	0.302	0.243	0.172	0.220	0.078						
Sometimes	0.105	**0.417**	0.316	**0.479**	**0.563**	**0.504**	**0.517**						
Frequently	0.007	0.066	0.047	0.136	0.212	0.165	0.393						
**LOOK FOR A JOB**													
Never	**0.785**	**0.846**	**0.787**	**0.409**	**0.717**	**0.519**	**0.427**	43.1	33.4	<0.001	<0.001	0.1	0.1
Rarely	0.148	**0.101**	0.146	0.221	**0.145**	0.218	**0.175**						
Sometimes	0.058	**0.048**	0.057	0.248	**0.116**	0.190	**0.288**						
Frequently	0.010	**0.005**	0.009	0.121	**0.022**	0.072	**0.110**						
**COMMUNICATE NEWS THEY BELIEVE TO BE OF INTEREST TO ALL**													
Never	**0.737**	**0.939**	**0.471**	0.079	**0.545**	0.041	0.255	104.6	45.7	<0.001	<0.001	0.4	0.3
Rarely	0.216	0.057	0.339	0.236	0.275	0.172	0.275						
Sometimes	0.045	0.004	0.173	**0.495**	0.145	**0.511**	**0.309**						
Frequently	0.000	0.000	0.016	0.189	0.035	0.276	0.161						
**SHARE STATE OF MIND**													
Never	**0.956**	**0.940**	**0.638**	0.181	**0.480**	0.019	0.082	95.2	74.0	<0.001	<0.001	0.5	0.4
Rarely	0.043	0.057	0.279	0.333	0.305	0.121	0.194						
Sometimes	0.000	0.003	0.079	**0.399**	0.180	**0.494**	**0.426**						
Frequently	0.000	0.000	0.004	0.087	0.034	0.365	0.299						
**SHARE LINKS OF INTERESTING WEBS**													
Never	**0.861**	**0.864**	**0.588**	0.091	**0.433**	0.021	0.048	111.3	71.6	<0.001	<0.001	0.5	0.4
Rarely	0.125	0.116	0.294	0.251	0.295	0.123	0.152						
Sometimes	0.014	0.020	0.112	**0.531**	0.254	**0.564**	**0.608**						
Frequently	0.000	0.000	0.005	0.127	0.017	0.291	0.192						
**COMMUNICATE IDEAS/REFLECTIONS**													
Never	**0.797**	**0.871**	**0.598**	0.065	0.314	0.004	0.036	116.0	79.0	<0.001	<0.001	0.6	0.4
Rarely	0.178	0.118	0.294	0.231	**0.368**	0.058	0.168						
Sometimes	0.029	0.010	0.104	**0.588**	0.275	**0.545**	**0.492**						
Frequently	0.000	0.000	0.003	0.115	0.043	0.391	0.304						
**INFORM ABOUT WHAT THEY ARE DOING AT THE TIME OF WRITING**													
Never	**0.983**	**0.767**	**0.656**	0.173	0.269	0.023	0.026	88.9	88.9	<0.001	<0.001	0.5	0.4
Rarely	0.017	0.179	0.266	0.322	0.284	0.131	0.100						
Sometimes	0.000	0.050	0.075	**0.417**	**0.361**	**0.517**	**0.468**						
Frequently	0.000	0.003	0.003	0.087	0.085	0.328	0.406						
**INFORM ABOUT BRANDS OR PRODUCTS THEY USE**													
Never	**0.920**	**0.968**	**0.946**	0.219	**0.656**	0.198	0.281	87.9	54.9	<0.001	<0.001	0.4	0.3
Rarely	0.077	0.031	0.053	**0.391**	0.226	**0.383**	0.265						
Sometimes	0.003	0.001	0.002	0.318	0.104	0.336	**0.334**						
Frequently	0.000	0.000	0.000	0.072	0.014	0.082	0.120						
**COMMENT ON ADS, AND PUBLICITY**													
Never	**0.920**	**0.999**	**0.998**	**0.355**	**0.819**	0.350	0.390	38.9	30.7	<0.001	<0.001	0.3	0.3
Rarely	0.075	0.001	0.001	0.354	0.170	**0.354**	**0.415**						
Sometimes	0.004	0.000	0.000	0.252	0.010	0.255	0.133						
Frequently	0.000	0.000	0.000	0.040	0.001	0.0409	0.062						

*Boldface indicates the most importance by segments*.

† *SP=Spain*.

‡ *NL = The Netherlands*.

Table 4 contains the clusters' profiles obtained for each country. The size and name assigned to the groups are shown at the top. Note that there are four groups, three are in Spain and in The Netherlands (Introvert, Versatile, and Expert-Communicator), and one that is only present in Spain (Novel). To complete the composition of the segments that were revealed, we have analyzed the profile of the resulting groups according to the information from the covariates introduced in the model. The information presented in Tables 4, 5 are the conditional probabilities about indicators and covariates respectively, that is, the differences in response patterns that distinguish each cluster.

**Table 5:** **Profile of latent segments (covariates)**.

**Descriptive Criteria**	**Categories**	**Introvert**		**Novel**	**Versatile**		**Expert-communic**.		**χ**^**2**^		***p*****-value**	
		**SP^†^**	**NL^‡^**	**SP**	**SP**	**NL**	**SP**	**NL**	**SP**	**NL**	**SP**	**NL**
Gender	Men	**0.646**	0.467	0.474	**0.606**	0.426	0.346	0.310	22.2	2.9	0.000	0.234
	Women	0.353	**0.533**	**0.526**	0.393	**0.574**	**0.654**	**0.690**				
Age	Less than 25	0.117	0.162	**0.278**	0.162	0.180	**0.403**	**0.341**	74.5	20.8	0.000	0.008
	From 25 to 29	0.091	0.102	0.227	0.169	0.148	0.273	0.068				
	From 30 to 35	0.104	0.168	0.144	**0.230**	**0.249**	0.182	0.159				
	From 36 to 44	0.221	0.192	0.227	**0.230**	0.185	0.091	0.250				
	Over 44	**0.468**	**0.377**	0.124	0.209	0.238	0.052	0.182				
Experience with SNS	Less than 1 month	**0.069**	**0.044**	0.039	0.036	0.035	0.008	0.045	35.1	10.0	0.000	0.124
	Between 1 and 6 mo.	0.314	0.109	**0.336**	0.277	0.094	0.136	0.047				
	Between 6 mo. and 1 year	0.196	0.137	0.269	0.252	0.071	0.276	0.200				
	Over 1 year	0.421	0.710	0.355	**0.435**	**0.800**	**0.580**	**0.708**				
Frequency of Participation in SNS	At least once a week	**0.685**	**0.666**	0.109	0.091	0.252	0.000	0.135	323.4	114.0	0.000	0.000
	Several times a week	0.095	0.162	**0.409**	0.334	**0.319**	0.070	0.212				
	At least once a day	0.019	0.137	0.194	**0.330**	0.299	0.293	**0.371**				
	Several times a day	0.000	0.035	0.288	0.245	0.129	**0.637**	0.281				
Time SNS used weekly^a^	Less than 1 h	**0.753**	**0.746**	0.241	0.281	0.372	0.103	0.116	195.4	143.3	0.000	0.000
	Between 1 and 5 h	0.247	0.248	**0.539**	**0.514**	**0.530**	0.353	0.453				
	More than 5 h	0.000	0.006	0.220	0.205	0.098	**0.544**	**0.431**				
Profile location in the SNS	Public	**0.401**	**0.228**	0.078	0.267	0.222	0.285	0.227	63.3	18.0	0.000	0.006
	Some private and others public	0.111	0.129	0.262	**0.434**	**0.263**	0.348	0.284				
	Private	0.317	0.504	**0.383**	0.237	0.425	**0.367**	**0.489**				
	Does not know	0.177	0.139	0.077	0.063	0.090	0.000	0.000				
Number of contacts	Less than 10	**0.392**	0.246	0.124	0.140	0.139	0.038	0.066	125.8	25.3	0.000	0.000
	From 10 to 50	0.495	**0.340**	0.485	**0.496**	0.257	0.373	0.224				
	From 51 to 100	0.093	0.216	0.225	0.247	0.294	0.249	0.234				
	More than 100	0.020	0.197	0.166	0.117	**0.311**	**0.340**	**0.476**				
Nature of contacts^b^	People known outside of Internet	0.379	**0.759**	0.784	0.750	**0.918**	0.798	**0.832**	55.6	17.4	0.000	0.000
	People known before but now only have contact through Internet	0.243	**0.620**	0.730	0.609	**0.716**	**0.798**	**0.746**	88.8	5.4	0.000	0.067
	People known through Internet with possibility of having real contact	0.051	0.046	0.108	**0.346**	0.166	0.244	0.152	80.6	12.7	0.000	0.002
	People known on the Internet with no possibility of real contact	0.026	0.117	0.097	**0.346**	0.235	0.229	0.178	78.2	7.7	0.000	0.022
Number of SNS they use	1	**0.485**	**0.659**	0.009	0.062	0.503	0.025	0.523	158.9	24.3	0.000	0.018
	2	0.331	0.233	**0.581**	0.422	**0.254**	0.364	0.204				
	3	0.169	0.084	0.225	**0.290**	0.127	0.276	**0.204**				
	More than 3	0.015	0.024	0.165	0.226	0.117	**0.334**	0.068				
Motives for using SNS	Entertainment	0.429	0.360	0.806	0.802	**0.584**	**0.888**	**0.683**	69.0	23.0	0.000	0.000
	Professional interest	**0.188**	0.163	0.099	**0.189**	0.174	0.148	**0.311**	5.2	5.9	0.160	0.052
	Because was invited	0.579	0.461	**0.666**	0.576	**0.507**	0.587	**0.502**	6.9	0.8	0.076	0.667
	Novelty	0.141	0.174	0.155	0.167	0.286	**0.312**	**0.400**	18.0	11.2	0.000	0.004
	Maintain contact with friends	0.329	0.496	0.734	0.635	**0.708**	**0.866**	**0.841**	68.7	24.4	0.000	0.000
	Because their friends were there	0.052	0.159	0.284	0.187	0.354	**0.434**	**0.588**	51.3	35.6	0.000	0.000
	Keep informed about events, parties, etc.	0.027	0.027	0.157	0.146	0.060	**0.505**	**0.270**	112.6	33.3	0.000	0.000
	Keep informed of comments on new products	0.079	0.035	0.011	0.103	0.085	**0.166**	**0.110**	20.5	5.0	0.000	0.081
	Make new friends	0.043	0.047	0.165	0.276	0.176	**0.306**	**0.443**	38.6	45.9	0.000	0.000
	Make contacts on a professional level	0.096	0.136	0.048	**0.201**	0.163	0.175	**0.299**	23.1	6.2	0.000	0.044
	Know better or have a closer relationship with persons with whom they do not have a direct relationship	0.027	0.060	0.209	0.174	0.114	**0.463**	**0.211**	86.2	8.7	0.000	0.013
	Look for partner/dating	0.027	0.028	**0.099**	0.075	0.049	0.089	**0.069**	4.0	1.5	0.257	0.477

*Boldface indicates the most relative importance by segments*.

† *SP = Spain*.

‡ *NL = The Netherlands*.

a *These intervals were estimated by the Latent Gold statistical program, as the variables introduced were numeric*.

b *Only positive values (yes) have been reflected in the Table*.

Table 5 shows the composition of each group based on the descriptive criteria included in the analysis.

Tests associated with chi-square statistic (χ2) conclude that significant differences exist between the segments for each country regarding gender, age, frequency of participation in SNS, weekly time spent on SNS, profile location on these sites, number of contacts, types of contacts maintained (in The Netherlands there are no significant differences with regard to people previously known but now only in contact through the Internet), and number of SNS in which the user has an account and is active. With regard to experience in SNS, there are only significant differences between the segments in Spain. For most of the motives that trigger the use of the SNS we also found significant differences in both countries, except for reasons such as the use of these sites for professional purposes, because the user was invited, and in the case of looking for a partner or for dating.

The main characteristics of the above-mentioned groups in both countries, listed from a lesser to a higher intensity of SNS use, are detailed below:

- Introvert User. This is the smallest group in Spain, representing 18.6% of SNS users, but in The Netherlands this group is bigger (41.3%). This is the least active group, using SNS mainly to send private messages. In other words, they use these sites as an email substitute, as well as for updating their profile, which they do not do very often. Moreover, this group in The Netherlands uses SNS to look for friends. This segment is mainly made up by men in Spain and by women in The Netherlands, with a high percentage being over 44 years old. These users connect to SNS with low frequency and they spend less time on them. Spanish Introvert Users usually have a public profile and Dutch users have a private profile, having both fewer than 50 contacts. Introvert Users have contacts that they know outside the Internet and people with whom they have maintained previous contacts offline. The majority use only one SNS mostly because they were previously invited, and the Dutch users also use it to contact friends and acquaintances.
- Novel User. This group is unique in Spain (25.2%). Once in a while they comment on their friends' photos, send private and public messages and make comments on what is said or done by the people who send them the pictures. They also update their profile, but with lower probability, they look for information of things of their interest, label friends in pictures, and browse through user profiles. The majority are women less than 29 years old. They participate several times a week in SNS, spending between 1 and 5 h weekly. The majority have a private profile, with less than 50 contacts that they have known outside of Internet and with whom they may or may not have physical contact at this moment. They have accounts in two SNS and use them mainly for entertainment, to keep in touch with friends and people they know and because they were invited.
- Versatile User. The largest group in both countries (36.2% in Spain and 47.4% in The Netherlands). A Versatile User carries out almost all activities, although not all with a high intensity. The majority in both countries share or upload photos, update their profile and send private messages. This group of users is more active in Spain than in The Netherlands. The majority of Spanish Versatile Users, moreover, comment on friends' photos, comment on what the people they follow are saying, send public messages, share interesting website links and communicate ideas/reflections. These activities are also carried out by the Dutch Versatile Users, but not by all of them. On the other hand, once in a while these users in both countries obtain information about things that interest them, look for friends and inform about what they are doing at the time of writing. Moreover, only Spanish Versatile Users occasionally browse across SNS and their users' profile, label friends in pictures, communicate news or subjects that they believe can be of interest to all and share their state of mind, and rarely download applications or games, or inform about brands or products that they use. This segment is made up mainly of men in Spain and women in The Netherlands, between 30 and 44 years old. They participate several times a week in SNS sessions, spending between 1 and 5 h a day. Spanish Versatile Users are users of two or three SNS, with both private and public profiles, and they have between 10 and 100 contacts. However, Dutch Versatile Users are users of one SNS, with a private profile, and they have more than 50 contacts. The majority of contacts in both countries are people they knew before, in a physical environment. The majority use SNS for entertainment, to maintain contact with friends, and because they were invited.
- Expert-Communicator User. This group covers 19.9% of the Spanish and 11.3% of Dutch SNS users. This is the most active user of all groups, in addition to having the greatest probability of carrying out activities similar to the Versatile User. They also comment on ads and publicity. Their most outstanding feature is that they have a high probability of frequently carrying out several activities, specifically sharing or uploading photos, sending private messages and obtaining information of their interest. Moreover, Spanish Expert-Communicator Users carry out other activities frequently, while Dutch Expert-Communicator Users do them less frequently: specifically, commenting on friends' photos, commenting on what the people they follow do/say, browsing across SNS and their users' profiles, updating their profile, sending public messages and labeling friends in pictures. The highest proportion in both countries is composed of women aged under 25, with quite a lot of experience in SNS. They are the most participative users within these sites. In Spain, this group is made up of users of more than two SNS, and in The Netherlands they are users of one SNS, although there are a high proportion of users with more than two accounts. Many of these users in both countries have a private profile, and a higher number of contacts than the other groups, mainly with people they know offline. They use SNS for entertainment, because they were invited and for keeping in touch with friends. Moreover, the majority of Spanish users uses SNS to keep informed about events, parties, etc., and the Dutch users because their friends were there. In addition, this group encompasses a greater proportion of individuals than the rest of the groups who use the sites because of their novelty, to keep informed of comments on new products that they are interested in, to make new friends and to know better or have a closer relationship with persons with whom they do not have a direct relationship. Also, in this group, there is a higher proportion than in other groups of Spanish users who use SNS to keep informed of comments on new products of their interest, and Dutch users looking for partners.

## Conclusions, discussion and future research

Social Media Networks provide people with bodies that are combinations of embodied and technologically mediated action. These tools create multiple visibility formats within the infospheres of social media. Goodings and Tucker (2013) explore how socially mediated bodies are disposed for action in ways that involve negotiating communication through the mediated noise of social media, along with managing bodies that are faced with the spatialisation of time through new features such as Facebook's Timeline.

The differences in the adoption and behaviors within SNS may be due both to personal characteristics and cultural differences. According to our theory review, there are strong motives to believe that there are connections between culture and the way people use specific information technologies (Straub et al., 1997). Duet to the globalization and corporate multi-nationalism, understanding the influence of culture might be of critical importance (Sun and Zhang, 2006).

Hofstede's cultural framework has been strongly supported empirically (Søndergaard, 1994) and has been recognized as one of the most influential theory of culture among social sciences scholars (Nakata and Sivakumar, 2001). If we compare the cultural dimensions proposed by Hofstede for Spain and The Netherlands, we can conclude that Spain is characterized by a higher power distance and higher uncertainty avoidance. The Netherlands is mainly characterized by individualism (at the same time, it has lower power distance and low uncertainty avoidance) and low masculinity (high femininity). Thus, clear differences can be observed between Spain and The Netherlands, mainly in the degree of individualism, power distance and uncertainty avoidance.

The analysis of these cultural differences can be useful both for academic and professional reasons. The comparative analysis of the profiles of users of this type of web app, are increasingly relevant to observe the homogeneity or heterogeneity of their online behavior and to guide e-marketing decisions by companies in countries with technologically different cultures (Kim et al., 2011).

Applying latent segmentation, in Spain, we have obtained four different user segments which have been classified as “Introvert,” “Novel,” “Versatile,” and “Expert-communicator.” However, in The Netherlands, we obtained only three groups that we have tagged as “Introvert,” “Versatile,” and “Expert-communicator.” These three segments are very similar to those obtained in Spain. In fact, we have call them the same. The results indicate that the socio-demographic characteristics by themselves are not adequate segmentation criteria for this context. More attention should be paid to criteria related to the use of SNS. The study reveals the different behavior of these segments, providing organizations with important information as a basis for designing strategies for the use of SNS as marketing tools.

Each group uses different SNS applications and with different frequencies. The “Introvert” uses these sites once in a while to communicate with their friends privately. In sum, the “Novel” user, which only exists in Spain, uses the SNS once in a while to maintain contact with their friends (obtaining and giving information about each other). The “Versatile” user carries out different activities, although only occasionally. However, the “Expert-communicator” users carry out a greater number of activities and with greater frequency, especially relating to bi-directional communication with their friends. In both countries, the “Expert-Communicators” present the most interesting possibilities as potential sources of market information and possibilities for engagement as brand ambassadors.

As we proposed in the introduction section, companies should understand the heterogeneity in consumer behaviors within these virtual social environments. Segmentation present numerous benefits allowing a greater customer satisfaction and higher level of customer loyalty. Therefore, it might be interesting for companies to identify “Expert-Communicators” segments and to attempt to increase the number of engaged customers and create brand advocates by better understanding the needs and motives of their socially networked customers. These consumers could be engaged in open dialogue, since they present an interesting possibility as potential sources of marketing information and for engagement as brand ambassadors. However, it is worth noting that this segment is not the only one with interest for marketing purposes, it depends on the objectives and the target of the company.

The information available in SNS, voluntarily uploaded by their users, allows companies to obtain a huge amount of information about their customers regarding their habits, personalities, and lifestyles. This information allows refined market segmentation. An analysis of users' behaviors can also provide an early warning of unknown product problems as companies will have a high valuable information about the customer preferences, complains…and also can help to detect some possible problems in the beginning stages of the product introduction in the market. By monitoring the comments and the information available in SNS, companies will have a highly valuable information to improve the knowledge of their customer needs and preferences. Therefore, businesses can use SNS as a source of customer voice. They can obtain, at very low cost, the direct and valuable market information that they need to improve decision-making, to monitor opinions and complaints about the organization, or even for providing suggestions about new products or services.

The paper proposes an integrated and interesting segmentation methodology to understand affective and behavioral dimensions of SNS users and can be extended to the analysis of other tools or technological devices. In that way, and a as future research we propose, for example, to extend the cross-cultural analysis focussed on the use of SNS through the mobile technologies (Aldás et al., 2010; Baron and Hard af Segerstad, 2010; Westlund, 2010). The cultural differences between countries (not only in the use of social networking sites but also in the use of mobile technologies) could be interesting to be taken into account by managers in their marketing decisions in order to have a better comprehension of their target markets.

### Conflict of interest statement

The authors declare that the research was conducted in the absence of any commercial or financial relationships that could be construed as a potential conflict of interest.
